# Co-culture and biogeography of *Prochlorococcus* and SAR11

**DOI:** 10.1038/s41396-019-0365-4

**Published:** 2019-02-11

**Authors:** Jamie W. Becker, Shane L. Hogle, Kali Rosendo, Sallie W. Chisholm

**Affiliations:** 1 Department of Civil and Environmental Engineering, Massachusetts Institute of Technology, Cambridge, MA USA; 2 Department of Biology, Massachusetts Institute of Technology, Cambridge, MA USA; 3 Present Address: Department of Biology, Haverford College, Haverford, PA USA

**Keywords:** Microbial ecology, Microbial biooceanography, Water microbiology

## Abstract

*Prochlorococcus* and SAR11 are among the smallest and most abundant organisms on Earth. With a combined global population of about 2.7 × 10^28^ cells, they numerically dominate bacterioplankton communities in oligotrophic ocean gyres and yet they have never been grown together in vitro. Here we describe co-cultures of *Prochlorococcus* and SAR11 isolates representing both high- and low-light adapted clades. We examined: (1) the influence of *Prochlorococcus* on the growth of SAR11 and vice-versa, (2) whether *Prochlorococcus* can meet specific nutrient requirements of SAR11, and (3) how co-culture dynamics vary when *Prochlorococcus* is grown with SAR11 compared with sympatric copiotrophic bacteria. SAR11 grew 15–70% faster in co-culture with *Prochlorococcus*, while the growth of the latter was unaffected. When *Prochlorococcus* populations entered stationary phase, this commensal relationship rapidly became amensal, as SAR11 abundances decreased dramatically. In parallel experiments with copiotrophic bacteria; however, the heterotrophic partner increased in abundance as *Prochlorococcus* densities leveled off. The presence of *Prochlorococcus* was able to meet SAR11’s central requirement for organic carbon, but not reduced sulfur. *Prochlorococcus* strain MIT9313, but not MED4, could meet the unique glycine requirement of SAR11, which could be due to the production and release of glycine betaine by MIT9313, as supported by comparative genomic evidence. Our findings also suggest, but do not confirm, that *Prochlorococcus* MIT9313 may compete with SAR11 for the uptake of 3-dimethylsulfoniopropionate (DMSP). To give our results an ecological context, we assessed the relative contribution of *Prochlorococcus* and SAR11 genome equivalents to those of identifiable bacteria and archaea in over 800 marine metagenomes. At many locations, more than half of the identifiable genome equivalents in the euphotic zone belonged to *Prochlorococcus* and SAR11 – highlighting the biogeochemical potential of these two groups.

## Introduction

The global ocean is numerically dominated by *Prochlorococcus* and SAR11 (*Pelagibacterales*). *Prochlorococcus*, a cyanobacterium, is the most abundant primary producer in tropical and subtropical waters, where its estimated 2.9 × 10^27^ cells produce ca. 4 Gt of organic carbon annually [1]. As such, they support a notable fraction of the secondary production in these nutrient-poor waters [2]. Members of the alphaproteobacteria known as SAR11 are found throughout the marine environment with an estimated global abundance of 2.4 × 10^28^ cells, about half of which are in the euphotic zone [3]. *Prochlorococcus* and SAR11 together have been estimated to comprise roughly 40–60% of the total bacteria in oligotrophic surface waters at Station ALOHA in the North Pacific [4].

Since its discovery three decades ago, *Prochlorococcus* has emerged as a powerful model organism for microbial ecology [5]. Its ecotype diversity is well-characterized [6–9] and there is an increasing wealth of genomic, transcriptomic, and proteomic information applied to this group [10–12]. The discovery of SAR11 a few years later further advanced our understanding of microbial ecology and evolution in the oligotrophic ocean [13, 14]. SAR11 possesses many traits that highlight adaptations to the oligotrophic marine environment, including a small genome size [15, 16] and unique metabolic dependencies [17–20] partitioned among diverse ecotypes with distinct biogeography [21–24].

In addition to having large population sizes consisting of genetically distinct ecotypes, or clades, *Prochlorococcus* and SAR11 have adapted to their oligotrophic habitat by minimizing their genome content and metabolic versatility. SAR11 and high-light adapted *Prochlorococcus* have small cell sizes between 0.2 and 0.8 microns in diameter, small genomes (1.2–1.8 Mb) with low guanine-cytosine content (29–32%GC) [10, 15], and fewer regulatory σ-factors than would be predicted from the size of their genomes [25]. Low-light adapted *Prochlorococcus* genomes are larger (ca. 2.5 Mb) with higher guanine-cytosine content (33–50%GC), but are still relatively streamlined compared to copiotrophic bacteria. Genome reduction has resulted in the loss of metabolic capabilities in both *Prochlorococcus* and SAR11, some of which, such as reduction of oxidative stress [26] or a requirement for exogenous reduced sulfur [17] respectively, are provided by neighboring microbes. Likely as a result of these types of interdependencies, both taxa are difficult to isolate and maintain in vitro compared to faster-growing (r-selected) microbes; many of the same traits that confer a competitive advantage in their native habitat likely render them difficult to maintain in a laboratory.

*Prochlorococcus* and SAR11 cells can interact with each other in the surface ocean either directly or indirectly through the exchange of metabolites. Like all heterotrophs, SAR11 cells rely on organic matter derived from primary producers and recent experiments revealed that some members of the SAR11 clade likely rely heavily on organic compounds as an important source of phosphorus in addition to carbon [27]. SAR11 also has several unique metabolic requirements that implicate potential mutualism with *Prochlorococcus*. For example, SAR11 exhibits auxotrophy for a thiamin precursor molecule found in spent medium from *Prochlorococcus* [20] and some strains have a glycine requirement that can be partially met by glycolate, a byproduct of *Prochlorococcus* photorespiration [2, 28]. Furthermore, evidence from comparative genomics and metabolic modeling suggests that *Prochlorococcus* and SAR11 may exchange glycolate, pyruvate, and malate through complementary and/or co-dependent metabolic pathways, and it is likely that the evolution of these two organisms has been tightly interwoven [29].

Despite the high abundances and frequently observed habitat overlap of *Prochlorococcus* and SAR11 in the tropical and subtropical oligotrophic ocean, a system for co-culturing and exploring interactions between members of these two model groups has remained elusive, largely due to their cryptic growth requirements and inherent challenges in maintaining laboratory isolates. Here we report the development of stable (over 2 year) co-cultures of *Prochlorococcus* and SAR11, and explorations into the interrelated growth dynamics of these groups. Specifically, we sought to determine whether *Prochlorococcus* can provide SAR11 with individual growth requirements and how SAR11/*Prochlorococcus* co-culture dynamics compare to co-cultures of *Prochlorococcus* and a suite of sympatric copiotrophic bacteria. We also report results from an updated global census, incorporating recently acquired metagenomic data that utilize a genome size-independent approach (genome equivalents) to highlight the collective dominance of *Prochlorococcus* and SAR11 in the surface ocean. The biogeography of *Prochlorococcus* and SAR11 provides a framework in which to understand the global impact potential of interactions between these groups. Co-cultures of *Prochlorococcus* and SAR11 constitute a novel and ecologically significant model system for the study of marine microbial ecology.

## Materials and methods

### Biogeography analysis

The computational steps for determining the genome equivalents of SAR11, *Prochlorococcus* and other identifiable bacteria and archaea are described in detail in supplementary methods. Briefly, we analyzed 195 surface, mixed layer, and deep chlorophyll maximum layer metagenomes from the Tara oceans project [30–32], 480 metagenome samples acquired during GEOTRACES cruises, and 133 metagenome samples from the HOT and BATS oceanographic time series [33]. Metagenome reads were Illumina adapter trimmed, quality filtered, and overlapped using the bbtools (BBMap V37.90) software suite [34]. We annotated the quality controlled metagenomes against a custom reference database of approximately 26,000 bacterial, archaeal, viral, and microbial eukaryotic isolate, single cell, and metagenome and transcriptome assembled genomes compiled from various sources [35–43] using Kaiju (V1.6.0; [44]). The taxonomic composition of the reference database was intended to predominantly reflect that of the marine environment, while minimizing (but not excluding) the representation of clinical, industrial, and terrestrial host-associated genomes/samples. The majority of reads in our study (54% ± 10%) could be classified across all metagenomes using this approach (see supplementary methods). We extracted the reads from each metagenome classified by Kaiju as *Prochlorococcus* (genus), SAR11 (order *Pelagibacterales*), and bacteria/archaea (kingdom), and then quantified universal, single-copy marker genes within each taxonomically resolved pool using MicrobeCensus (V1.1.1; [45]). The abundances of these marker genes were used to estimate ‘genome equivalents’ (the operational number of genomes represented by single-copy marker genes) within each taxonomically resolved read pool. We report abundances as marker-gene resolved genome equivalents rather than total classified reads due to systematic variations in the average genome size between groups like *Prochlorococcus* and SAR11 and the rest of the microbial community [45]. Here, the relative abundance of *Prochlorococcus* or SAR11 is defined by the fraction of *Prochlorococcus* or SAR11 genome equivalents divided by the total number of bacterial and archaeal genome equivalents that could be identified in each sample. Phylogenies presented in the supplemental material are derived from a concatenated protein multiple sequence alignment based on 120 taxonomically conserved single-copy marker genes [41, 46] and were inferred with RAxML (V8.2.9; [47]).

### Strain selection and isolation

*Prochlorococcus* strains were chosen to represent clades with genetic and physiological distinctions that influence their biogeographic distributions [10, 48] with a preference for strains isolated from the N. Atlantic Ocean, the place of origin of *Pelagibacterales sp*. HTCC7211 (Table 1). HTCC7211 was isolated in 2006 from 10 m at the Bermuda Atlantic Time-series Study site in the Sargasso Sea and is a member of the abundant warm-water surface-dwelling Ia.3 ecotype [24] (Fig. S1). Several new strains of *Prochlorococcus* and heterotrophic bacteria were isolated to examine interactions between isolates from the same water sample – i.e. sympatric strains. Details regarding their isolation are included in Table 1 and supplementary methods.

**Table 1:** Isolates used in this study. The phylogenetic affiliations of the *Prochlorococcus* and SAR11 strains are shown in Fig. S1

*Prochlorococcus*					
Strain	Clade	Isolation location	Depth (m)	Year	Reference
MED4	HLI	Mediterranean Sea	5	1989	[75]
MIT9312	HLII	Gulf Stream	135	1993	[76]
MIT9301	HLII	Sargasso Sea (BATS)	90	1993	[77]
MIT1314	HLII	North Pacific (Station ALOHA)	150	2013	This study
MIT0801	LLI	Sargasso Sea (BATS)	40	2008	[78]
MIT9313	LLIV	Gulf Stream	135	1993	[76]
MIT1320	LLIV	North Pacific (Station ALOHA)	150	2013	[79]
MIT1327	LLIV	North Pacific (Station ALOHA)	150	2013	[79]
*Heterotrophic bacteria*					
Strain	Order (clade/genus)	Isolation location	Depth (m)	Year	Reference
HTCC7211	SAR11 (*Pelagibacterales*)	Sargasso Sea (BATS)	10	2006	[80]
MIT1351	*Rhodospirillales* (*Thalassospira*)	North Pacific (Station ALOHA)	150	2013	This study
MIT1352	*Rhodobacterales* (*Roseobacter*)	North Pacific (Station ALOHA)	150	2013	This study
MIT1353	*Alteromonadales* (*Marinobacter*)	North Pacific (Station ALOHA)	150	2013	This study

### Development of ProMS medium for growing both *Prochlorococcus* and SAR11

While media recipes exist for culturing both *Prochlorococcus* [49] and SAR11 [28, 50], none that we tested could support the growth of both strains. To this end, we established ProMS, a medium with a 0.2 µm filtered, autoclaved Sargasso surface seawater base, that was capable of supporting the growth of each strain in both mono- and co-culture (Table S1). After autoclaving, the seawater was sparged with sterile CO_2_ followed by air to reestablish a bicarbonate-based buffer system [51] and amended with sterile Pro99 nutrients [49] and organic compounds to meet the known unique metabolic requirements of SAR11 [52] as follows: pyruvate (1 µM), glycine (1 µM), methionine (0.2 µM), and the vitamin mix developed for AMS1 medium [28] diluted 50-fold. Organic additions were modeled after the medium developed by [28], with reduced concentrations designed to promote interactions.

In preparation for co-culture experiments, axenic SAR11 cells were first transferred from AMS1 medium amended with pyruvate (50 µM), glycine (50 µM) and methionine (10 µM) to ProMS medium. Prior to co-culturing experiments, monocultures of *Prochlorococcus* and SAR11 were maintained using ProMS medium in acid-washed autoclaved polycarbonate tubes at 22 °C under constant illumination (12 µmol photons m^−2^ s^−1^) for >25 consecutive transfers (>100 generations).

### Co-cultures of *Prochlorococcus* and SAR11

Following acclimation of individual strains to ProMS medium, triplicate 6 ml batch co-cultures of SAR11 and diverse strains of *Prochlorococcus* were established at an initial ratio of 10:1 *Prochlorococcus*:SAR11 in acid-washed autoclaved polycarbonate tubes (10 ml capacity). Co-cultures were monitored for growth and purity by flow cytometry until both populations entered stationary and/or death phase.

### Organic nutrient substitution experiments

Monocultures of SAR11 and co-cultures of SAR11 and *Prochlorococcus* strains MED4 and MIT9313 were conditioned (where possible) onto versions of ProMS medium lacking either pyruvate, glycine or methionine to examine the ability of *Prochlorococcus* to supply SAR11 with specific nutrient requirements. SAR11 exhibited no change in maximum cell abundance in ProMS lacking pyruvate (likely due to the availability of compounds present in the Sargasso seawater base that can meet the central carbon requirement of SAR11), so the concentrations of glycine, methionine and vitamins in ProMS were increased 50-fold to create ProMC medium and induce pyruvate limitation.

### Co-cultures of *Prochlorococcus* and sympatric copiotrophs

For experiments with sympatric *Prochlorococcus* and heterotrophs, following acclimation of axenic strains to Pro99 medium, triplicate pairwise 6 ml batch co-cultures of *Prochlorococcus* strains MIT1314 and MIT1327 and heterotrophic bacteria strains MIT1351, MIT1352 and MIT1353 were established at an initial ratio of either 150:1 or 300:1 *Prochlorococcus*:heterotrophic bacteria for co-cultures involving MIT1314 and MIT1327 respectively (Table 1). Mono- and co-cultures were maintained in acid-washed autoclaved borosilicate glass tubes (10 ml capacity) and monitored for growth and purity as described above.

### Enumeration of cells

Cell concentrations were determined using a Guava Technologies easyCyte 12HT flow cytometer (EMD Millipore) after staining with SYBR green I (Lonza) in the dark for at least 55 min. Samples were diluted in sterilized Sargasso seawater to ensure <500 cells µl^−1^ to avoid coincidence counting. Samples were run with only the blue (488 nm) excitation laser enabled for maximum power and populations were resolved based on their green (525/30) and red (695/50) emission parameters.

## Results and discussion

### Co-occurrence of *Prochlorococcus* and SAR11 in the wild

We leveraged the recent expansion of marine metagenomic data to perform an updated global census of the *Prochlorococcus* genus and the *Pelagibacterales* order (hereafter SAR11) by estimating their relative abundance throughout the global ocean. We examined 668 shotgun metagenomes with a broad geographic distribution collected within the euphotic zone [30–33] and 133 metagenomes from the Bermuda Atlantic Time-series (BATS) and Hawaii Ocean Time-series (HOT) [33]. Total metagenome recruitment combined with the enumeration of single-copy marker genes was used to estimate the contribution of each group to the total identifiable bacterial and archaeal genome equivalents in each sample (see supplementary methods for additional information).

**Fig. 1:**
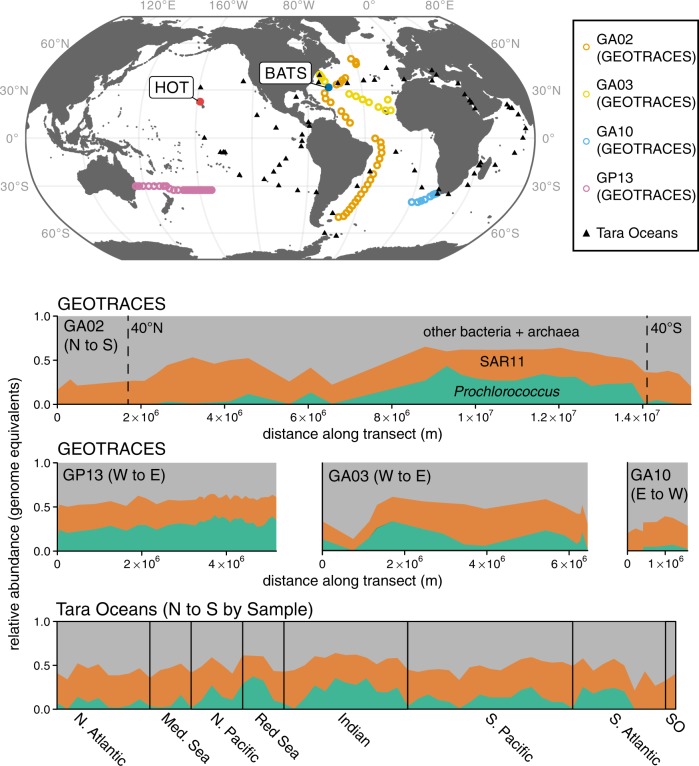
Genome equivalents of *Prochlorococcus* and SAR11 relative to total identifiable bacteria and archaea in the surface ocean. (Upper) Locations of GEOTRACES, HOT and BATS Time-series, and Tara Oceans metagenome samples used for the analysis. (Lower) Vertical axes represent the abundance of *Prochlorococcus* (green) and SAR11 (red) genome equivalents relative to all other identifiable bacteria and archaea (gray) throughout the upper 50 m of the global ocean. GEOTRACES horizontal axes depict the distance along each transect, while the Tara Oceans horizontal axis displays evenly spaced samples organized by latitude (N to S) within each oceanic region. Forty degrees N/S are marked on the GA02 panel

The relative abundance of SAR11 ranged between 7 and 55% (Fig. 1). Their contribution was largely horizontally and vertically consistent in the euphotic zone, although somewhat lower at high latitudes and coastal sampling sites, and in deeper waters near the base of this zone (Fig. 1 and S3). In contrast, the distribution of *Prochlorococcus* was bounded by 45 degrees N/S; its relative abundance began to decline sharply near 40 degrees N/S, consistent with previous reports using other approaches [1, 8]. The relative abundance of *Prochlorococcus* ranged from undetectable to >45% in parts of the remote south Pacific Ocean (Fig. 1). The contribution of *Prochlorococcus* generally decreased with depth and light intensity and its maximum was typically found in the upper 100 meters (Fig. S2). Between 40 degrees N/S in the upper 300 meters, the median ratio of *Prochlorococcus* to SAR11 genomes was 0.73, or roughly 3 *Prochlorococcus* cells for every 4 SAR11 cells (if one makes the large assumption that genome equivalents correspond directly to cell counts). The standard deviation of this value was large however (0.49), indicating high variability in the ratio of these groups in the wild - from about 1:5 to 2:1 *Prochlorococcus*:SAR11.

**Fig. 2:**
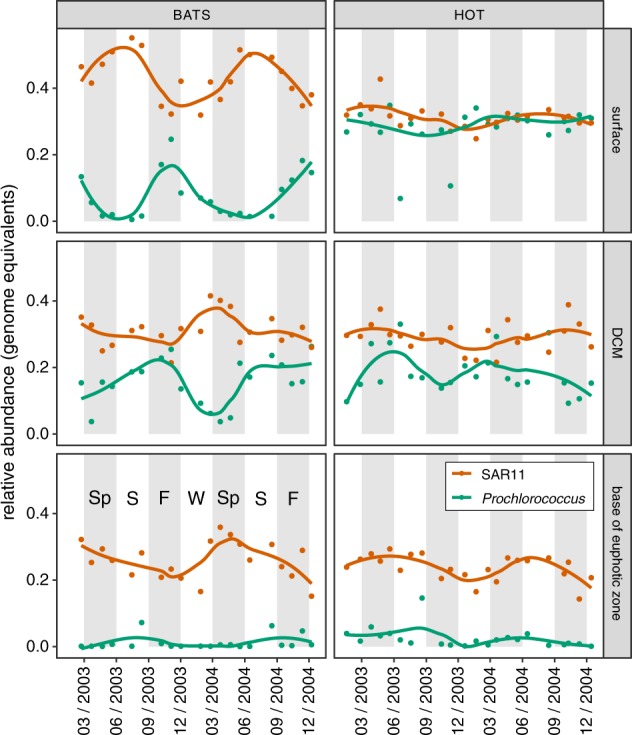
Seasonality of *Prochlorococcus* (green) and SAR11 (red) estimated genome equivalents in metagenomes sampled from two ocean time series sites - the Bermuda Atlantic Time-series (BATS) and Hawaii Ocean Time-series (HOT). Vertical axes represent the abundance of each group (genome equivalents) relative to total identifiable bacteria and archaea genome equivalents, while horizontal axes represent time (month/year). Data are faceted by time series (horizontal) and depth (vertical). Surface samples are from within the mixed layer (≤25 m), DCM corresponds to the depth of maximum chlorophyll a fluorescence, and the base of the euphotic zone is defined by the depth at which ca. 1% of surface photosynthetically active radiation remains (see [33] for details). Solid lines represent a local weighted regression analysis (LOESS) smooth function for each taxonomic group at each depth. Anomalously low abundances of *Prochlorococcus* are sporadically observed in surface waters at the HOT study site

Both *Prochlorococcus* and SAR11 are known to display seasonal growth dynamics [7, 53] – also evident here in metagenomes from the HOT and BATS time series sites (Fig. 2). In surface waters and the deep chlorophyll maximum (DCM) layer at the BATS site, the relative abundance of *Prochlorococcus* varied from nearly undetectable during the spring, to over 20% of the identifiable bacterial and archaeal community in late autumn, as the depth of the mixed layer increased – consistent with previous reports [7]. In contrast, the relative abundance of SAR11 peaked when the mixed layer depth shoaled to its most shallow depth, consistent with previously reported patterns for members of the SAR11 Ia clade [23]. The relative abundances of *Prochlorococcus* and SAR11 displayed less seasonality in surface waters at station ALOHA, but did undergo sporadic oscillations (e.g. June 2003) that may reflect mesoscale events such as nutrient influxes, temperature shifts, water transport, or entrainment caused by eddies [54]. In summary, the combined genome equivalents of *Prochlorococcus* and SAR11 regularly comprised more than half of the total identifiable bacteria and archaea in the euphotic zone of the oligotrophic tropical and subtropical ocean. We stress however, that metagenome-derived genome equivalents may not directly correspond to concentrations of viable cells, and furthermore, should not be interpreted as representing relative biomass, as these are among the smallest bacteria in the marine environment.

### Co-culture of *Prochlorococcus* and SAR11

Motivated by the overlapping biogeography of *Prochlorococcus* and SAR11 and their near ubiquity and high relative abundances in the surface ocean, we explored interactions between members of these groups by developing co-cultures of *Pelagibacterales sp*. HTCC7211 and a number of *Prochlorococcus* strains representing high-light and low-light adapted clades (Table 1). HTCC7211 (hereafter “SAR11”) was isolated from surface waters in the Sargasso Sea [55], making it an ideal candidate for co-culture with several of the *Prochlorococcus* strains isolated from the same location (Table 1). Through many rounds of trial and error, we designed a natural seawater-based medium (ProMS) that could support the growth of both cell types independently (Table S1). The growth rate of SAR11 increased from 0.35 d^−1^ (*s* = 0.01 d^−1^) when grown in AMS1 medium to 0.41 d^−1^ (*s* = 0.01 d^−1^) after acclimation to ProMS; however, the maximum cell abundance decreased from 1.1 × 10^8^ cells ml^−1^ to 7.5 × 10^6^ cells ml^−1^ (Fig. S3) due to the decrease in organic nutrient concentrations. The transfer of axenic *Prochlorococcus* from Pro99 to ProMS medium did not change its growth rate or maximum cell abundance.

**Fig. 3:**
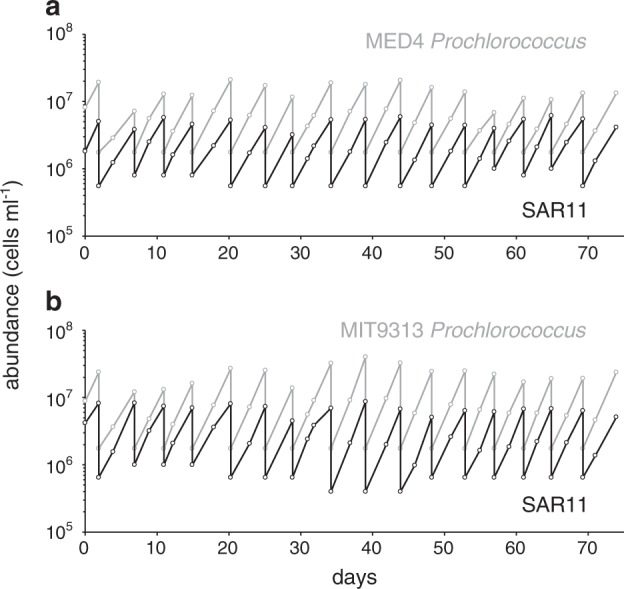
Cell abundance as a function of time in log-phase, semi-continuous batch co-cultures of SAR11 (*Pelagibacterales sp*. HTCC7211) (black lines) and *Prochlorococcus* strain MED4 (**a**; gray line) and MIT9313 (**b**; gray line) in ProMS medium. Dilution frequency and volume were dictated by *Prochlorococcus* cell density (see text)

We then propagated the strains in semi-continuous co-culture, where they remained stable for at least 2 years (Fig. 3 and S4). The frequency and magnitude of culture dilution was determined from experience, based on the cell concentrations of *Prochlorococcus*: if they exceeded ca. 5 × 10^8^ cells ml^−1^, the culture would transition into stationary phase and the SAR11 population would decline rapidly (see below). If diluted below 10^6^ cells ml^−1^, *Prochlorococcus* would display a lag phase or not grow at all – especially low-light adapted strains. Once stabilized within these limits, *Prochlorococcus* remained in log-phase growth if diluted to 1.25 × 10^6^ cells ml^−1^ every 3–4 days. Because dilution metrics were dictated solely by the density of *Prochlorococcus*, there was no reason *a priori* that SAR11 abundances would remain in quasi-steady state. At monoculture growth rates, SAR11 densities would have been diluted down to <1 cell ml^−1^ within 3 months using these dilution rates. That they reached a quasi-steady state when grown with *Prochlorococcus* implies that the metabolisms of the autotroph and heterotroph became coupled in some way, resulting in a relatively constant *Prochlorococcus* to SAR11 cell ratio of 3:1 throughout the co-culture period. We note that, although close, this ratio falls outside of the range we observe in the wild, a likely consequence of the myriad differences between nature and our laboratory cultivation conditions, including the absence of other organisms and replete concentrations of inorganic nutrients provided for robust *Prochlorococcus* growth in ProMS medium.

### *Prochlorococcus* and SAR11 growth dynamics in batch co-cultures

**Fig. 4:**
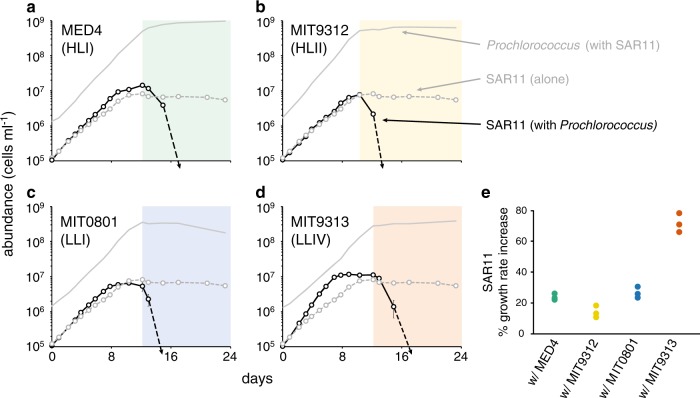
Growth of *Prochlorococcus* strains MED4 **a**, MIT9312 **b**, MIT0801 **c** and MIT9313 (**d**; solid gray lines in a-d reproduced from Fig. S5) and SAR11 (*Pelagibacterales sp*. HTCC7211) (black lines) in co-culture compared to the growth of SAR11 monocultures (dashed gray lines). Growth curves for *Prochlorococcus* monocultures are presented in Fig. S5. Shaded regions denote the interval when *Prochlorococcus* are in stationary phase. SAR11 cells were undetectable in co-culture with *Prochlorococcus* after day 12–15 (dashed black arrows). Circles represent the mean (±s.d.) of biological triplicates. Error bars are smaller than the size of the symbols where not visible. Dot plot **e** depicts percent increases in SAR11 growth rate due to the presence of each *Prochlorococcus* strain. The growth rate of SAR11 alone was 0.41 day^−1^

To examine these interactions in detail, SAR11 was co-cultured with 4 strains of *Prochlorococcus* representing two high-light adapted (HL) and two low-light adapted (LL) clades (Fig. S1, Table 1). SAR11 and *Prochlorococcus* cells were taken from early log-phase axenic cultures, inoculated into co-culture, and their growth patterns were monitored in mono- and co-culture. The growth of *Prochlorococcus* was not significantly influenced by the presence of SAR11 over the entire growth curve (Fig. S5). SAR11 however, grew 15–70% faster (depending on the *Prochlorococcus* strain) in the co-cultures than it did when grown alone (Fig. 4). SAR11 always entered stationary phase earlier than *Prochlorococcus* in the co-cultures, reaching maximum cell abundances that were at, or slightly above (up to twofold higher) those attained in monoculture (Fig. 4).

Precisely when *Prochlorococcus* entered stationary phase (beginning of colored shading in Fig. 4), SAR11 cell concentrations declined abruptly to below detection limits – in striking contrast to its behavior when grown alone where cell numbers simply level off in stationary phase (Fig. 4). The antagonistic effect of the co-culture conditions on SAR11 was apparent for all *Prochlorococcus* strains, revealing a condition-dependent shift likely caused by a growth phase-specific release of metabolites from *Prochlorococcus*, as has been observed in eukaryotic phytoplankton [56, 57]. Certain amino acids and osmolytes at high concentrations have been shown to slow or even prohibit the growth of this SAR11 strain [18, 58], providing targets for further metabolomic exploration. Carini et al. observed increases in DNA fluorescence due to the presence of elongated cell doublets for a different SAR11 strain (HTCC1062) experiencing pyruvate limitation, and went on to show that both the ratio of pyruvate:glycine and the concentration of alanine affected the degree of doublet formation [28]. Interestingly, we observe a similar increase in DNA fluorescence for HTCC7211 in co-culture with *Prochlorococcus* occurring at the precise moment when *Prochlorococcus* populations cease logarithmic growth and begin transitioning to stationary phase (Fig. S6). This suggests that as *Prochlorococcus* slows its growth rate, it may be impacting nutrient stoichiometry in a manner that disrupts the central carbon metabolism of SAR11. The concept of a dependence on specific nutrient ratios for efficient metabolic functioning and regulation has been presented as a possible consequence of genome streamlining in microbes such as SAR11 [25].

### *Prochlorococcus* co-cultured with sympatric copiotrophic bacteria

Although all but one of the *Prochlorococcus* strains discussed above were isolated from the same ocean as the SAR11 strain, they were not isolated from the same water sample; our attempts to isolate truly sympatric *Prochlorococcus* and SAR11 failed. We did, however, isolate two new *Prochlorococcus* strains (MIT1314 - HLII clade, and MIT1327 - LLIV clade, see Fig. S1, Table 1) along with a number of sympatric copiotrophic heterotrophic bacteria – i.e. strains that grow rapidly in media rich in organic matter. These heterotrophic strains have genomic characteristics (4.4 Mb; 55–59%GC) typical of copiotrophic bacteria [59] and represent taxa found at low abundances in the oligotrophic ocean, except when subjected to episodic disturbances [60]. We studied 3 of these – *Thalassospira sp*. MIT1351, *Roseobacter sp*. MIT1352, and *Marinobacter sp*. MIT1353 –in co-culture with sympatric *Prochlorococcus* to compare with SAR11 patterns (Table 1).

In contrast to SAR11, which would not grow alone in Pro99 medium [49] unless amended with labile substrates to meet its unique metabolic requirements (see Methods, Table S1), the copiotrophic strains grew appreciably in unamended Pro99 medium, apparently using their more diverse metabolic repertoire to subsist on residual organic carbon in the natural seawater base of the medium. Thus we used unamended Pro99 medium in this set of experiments. Similar to the results with SAR11, the presence of the copiotrophic bacteria did not greatly influence the growth of *Prochlorococcus* cultures during log phase. The presence of *Marinobacter* MIT1353 did, however, increase the growth rate of *Prochlorococcus* MIT1314 slightly (from 0.64 d^−1^ to 0.68 d^−1^) and the presence of all three copiotrophs resulted in slightly higher maximum densities of MIT1314 relative to growth alone (Fig. S7). In addition, *Prochlorococcus* MIT1327 was somewhat rescued from declining cell numbers in death phase by all three of the copiotrophic strains (Fig. S7). Similar enhancements have been observed before [61, 62] and are usually attributed to the ability of catalase-containing bacteria to detoxify reactive oxygen species in their surrounding environment [26, 63]. This mechanism may also play a role here, as all three copiotrophic strains possess the gene necessary for catalase production. However, SAR11 HTCC7211 also possesses a catalase gene, yet it had no effect on *Prochlorococcus* growth (Fig. S5). It is unclear why, but copiotrophic catalase mutants have also been shown to rescue the growth of *Prochlorococcus* from low densities, and the addition of exogenous catalase alone cannot replicate co-culture responses [64]. Furthermore, transcriptional studies of co-cultures suggest interactions between *Prochlorococcus* and copiotrophic bacteria beyond those related to oxidative stress [65, 66]. Our results support the notion that diverse copiotrophic bacteria facilitate *Prochlorococcus* growth and that these benefits are due in part to factors other than the production of catalase.

**Fig. 5:**
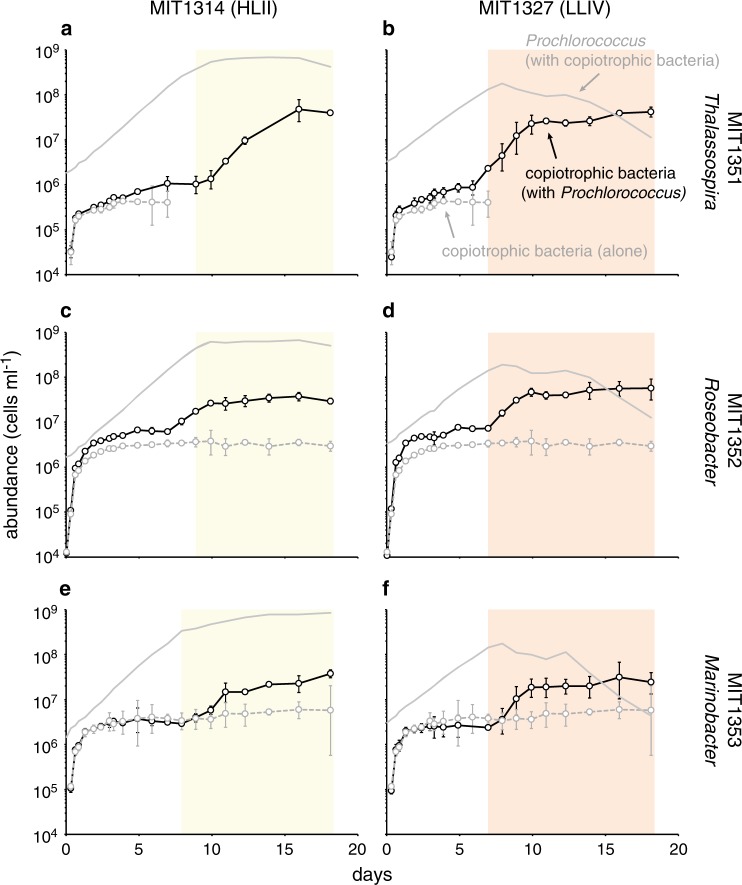
Growth of *Prochlorococcus* (solid gray lines, reproduced from Fig. S7) and copiotrophic bacteria (black lines) in co-culture compared to the growth of copiotrophic bacterial monocultures (dashed gray lines). Growth curves for *Prochlorococcus* monocultures are presented in Fig. S7. Shaded regions denote the interval when *Prochlorococcus* are in stationary phase. *Thalassospira* MIT1351 monocultures were indistinguishable from instrument noise after day 7, precluding reliable determination of cell concentrations. Circles represent the mean (±s.d.) of biological triplicates. Error bars are smaller than the size of the symbols where not visible

The response of the three copiotrophic bacteria to the presence of *Prochlorococcus* was strikingly different from that of SAR11, especially given that there are no added organic compounds in Pro99 medium. All three strains had an initial phase of rapid logarithmic growth (growth rate = 5.0–7.7 d^−1^; about an order of magnitude faster than that of SAR11), whether alone or in the presence of *Prochlorococcus*, presumably consuming organic matter present in the natural seawater base of the medium (Fig. 5). *Thalassospira* MIT1351 and *Roseobacter* MIT1352 reached higher cell abundances before entering stationary phase when grown with *Prochlorococcus* compared to growth alone (Fig. 5a–d), while this was not the case for *Marinobacter sp*. MIT1353 (Fig. 5e, f). In stark contrast to the SAR11/*Prochlorococcus* co-cultures, all three copiotrophs pulled out of stationary phase and resumed growth (at a rate of 0.49–0.82 d^−1^) when *Prochlorococcus* populations entered stationary phase (Fig. 5 shaded regions), displaying a diauxic growth pattern. This suggests that conditions produced by *Prochlorococcus* that are toxic to an oligotroph (e.g. SAR11) are ideal for opportunistic bacteria such as these, with more diverse functional repertoires and regulatory capabilities. Comparative genomic analysis reveals that all three copiotrophic bacteria possess more genes related to sugar catabolism, hydrolysis and beta-oxidation of fatty acids, binding of extracellular solutes, and transcriptional regulation than SAR11 HTCC7211, highlighting the contrasting degrees of metabolic flexibility among these strains (Table S2).

As mentioned above, a caveat in comparing these two sets of experiments is that the co-culture medium used in the SAR11 experiments was augmented with pyruvate, glycine, methionine, and pico- to nanomolar concentrations of 9 vitamins (Table S1), whereas the medium in the copiotroph experiments was unamended with organics. Despite this difference, the comparison is illuminating, given that the organic additions cannot explain the abrupt decline in SAR11 populations when *Prochlorococcus* enters stationary phase. The amensal phenotype with SAR11 was also observed in co-cultures with *Prochlorococcus* lacking exogenous pyruvate and glycine additions (data not shown), providing further support that these organics are not contributing to the disparate responses of SAR11 and copiotrophic bacteria in the co-cultures.

### *Prochlorococcus* can fulfill the central carbon requirement of SAR11

**Fig. 6:**
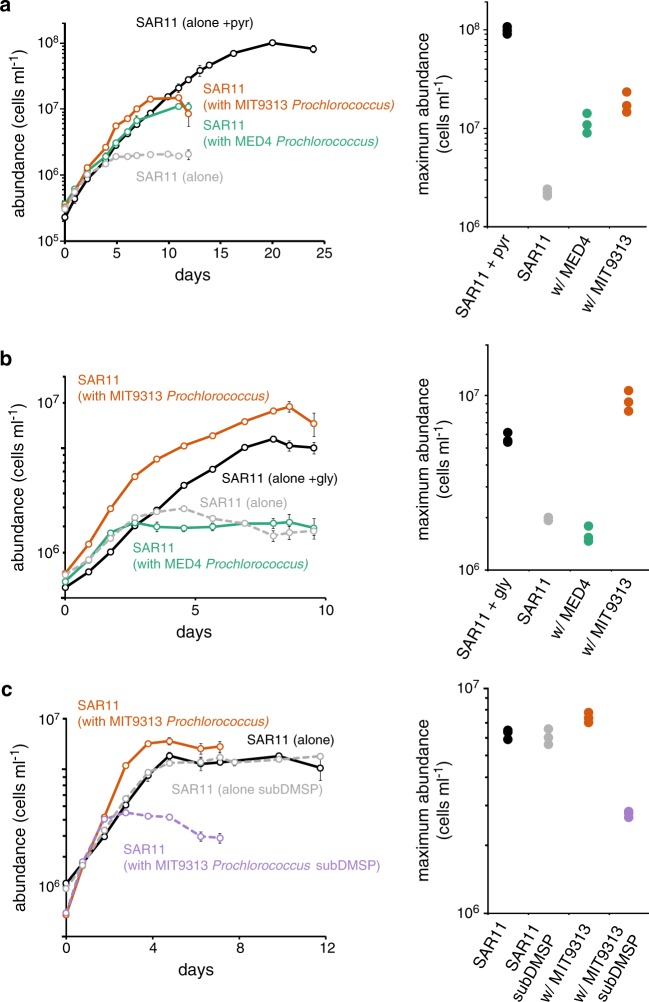
Growth of SAR11 (*Pelagibacterales sp*. HTCC7211) monocultures in the presence (black lines) and absence (dashed gray lines) of pyruvate **a** and glycine **b** compared to growth of SAR11 in co-culture with *Prochlorococcus* strains MED4 (green lines) and MIT9313 (red lines) in the absence of pyruvate **a** and glycine **b**. Panel **c** shows the growth of SAR11 monocultures in the presence of methionine (black line) or 3-dimethylsulfoniopropionate (DMSP; dashed gray line) compared to growth of SAR11 in co-culture with MIT9313 in the presence of methionine (red line) or DMSP (dashed violet line). Circles represent the mean (±s.d.) of biological triplicates. Error bars are smaller than the size of the symbols where not visible. Dot plots on the right depict the maximum cell densities obtained for each treatment. Dots are colored to match the corresponding lines in the adjacent growth curves

Low-molecular weight organic acids, including pyruvate, lactate, oxaloacetate, acetate, and taurine are central carbon sources for SAR11, including non-glycolytic strains like HTCC7211 [19, 28]. To determine whether *Prochlorococcus* can provide a central carbon source to SAR11, we grew the latter with two strains of *Prochlorococcus* –MED4 (HLI clade) and MIT9313 (LLIV clade) – in a medium with no added pyruvate (ProMC). Pyruvate is thought to be an essential central carbon compound available to all SAR11 [52, 67] and it was removed in order to drive the system toward pyruvate starvation. To ensure pyruvate limitation we increased the concentration of all other organic compound additions in ProMC (i.e. glycine, methionine, and 9 vitamins) 50-fold above ProMS levels, matching concentrations used to achieve maximum SAR11 densities [28]. A treatment with pyruvate added (50 µM) to ProMC served as a positive control. While the maximum abundance of SAR11 was reduced nearly 50-fold compared to the positive control when grown alone in ProMC, it was reduced only sevenfold and ninefold, respectively, in co-culture with MIT9313 and MED4. This could not be attributed to pyruvate carryover, as these results were obtained after extended semi-continuous culturing in ProMC over several months to remove any residual pyruvate (Fig. 6a). We conclude that both strains of *Prochlorococcus* produce and release organic matter that fulfills the central carbon requirement of SAR11.

What is *Prochlorococcus* supplying to SAR11 in these experiments? There are several candidate compounds. *Prochlorococcus* cells have been shown to release 9–24% of the inorganic carbon they assimilate as dissolved organic carbon, 4–20% of which is thought to be in the form of low molecular weight carboxylic acids, including glycolate, acetate and perhaps lactate [2]. Acetate and lactate can replace pyruvate for SAR11 growth [19]. Pyruvate is also predicted to be exported by *Prochlorococcus* as part of a strategy to maintain the cell’s redox balance [29]. Based solely on the SAR11 abundance data, it appears that *Prochlorococcus* MIT9313 provides additional labile central carbon substrates to SAR11on a per cell basis compared to MED4.

### *Prochlorococcus* MIT9313, but not MED4, can meet the glycine requirement of SAR11

SAR11 cells lack canonical genes for the biosynthesis of serine and glycine and instead rely on an exogenous source of these amino acids or their precursors [18]. To test whether *Prochlorococcus* cells can meet this unique SAR11 requirement, we removed glycine from the ProMS medium (ProMS -gly) and monitored the growth of SAR11 monocultures and co-cultures with *Prochlorococcus* strains MED4 or MIT9313 after several months of semi-continuous culture to remove any traces of glycine carryover. While SAR11 monocultures were able to grow in the ProMS -gly medium - indicating that the Sargasso seawater base supplied a source of glycine, serine, or precursors of these amino acids - its maximum abundance was fourfold lower than when grown in monoculture with 1 µM glycine. Co-culture with MED4 had no effect on this low yield, indicating that it did not provide a source of glycine or glycine substitutes to SAR11 (Fig. 6b). When in co-culture with MIT9313; however, the maximum abundance of SAR11 equaled or slightly surpassed that observed in monocultures with 1 µM glycine added. Thus, MIT9313, but not MED4, can fulfill the glycine requirement of SAR11.

It was initially surprising to us that MED4 was unable to meet SAR11’s glycine requirement, given that glycolate has been shown to substitute for glycine in SAR11 cultures [28] and glycolate production has been reported for MED4 in quantities that should have been sufficient to show an effect in our experiments [2]. The strain of SAR11 used by Carini et al (2013) was HTCC1062 (Ia.1 clade) however, and that used in our studies was HTCC7211 (Ia.3 clade). As it turns out, while HTCC1062 has the genes necessary to transport glycolate into the cell (*SAR11_0274*) and convert it to glyoxylate (*glcDEF*), a precursor of glycine [28], HTCC7211 does not, thus explaining why glycolate production by MED4 could not replace glycine in our experiments.

The most likely compound driving these disparate responses is the osmolyte glycine betaine, which has also been shown to substitute for glycine in SAR11 HTCC1062 [18, 28]. SAR11 HTCC7211, the strain used in our experiments, has all the genes necessary for the uptake of glycine betaine and its conversion to glycine [68]. *Prochlorococcus* MIT9313, and other members of its LLIV clade, have the genes responsible for the biosynthesis of glycine betaine (*gbmt1/2*), along with three genes that encode an ABC transporter for this molecule (*proVWX*) [69]. Accumulation of glycine betaine has been reported in MIT9313 [70] and a recent study has shown that this accumulation can be quite significant – up to 20% of the cell’s dry weight (K. Longnecker, E. Kujawinski, personal communication). Production of glycine betaine by *Prochlorococcus* is restricted to the LLIV clade and is thus absent in MED4, further supporting the hypothesis that glycine betaine production by LLIV *Prochlorococcus* cells can fulfill the glycine requirement for SAR11 growth.

### *Prochlorococcus* and the reduced sulfur requirement of SAR11

SAR11 cells do not contain the full complement of genes necessary for assimilatory sulfate reduction, thus they rely on the production and release of reduced organic sulfur compounds such as methionine, methanethiol, or 3-dimethylsulfoniopropionate (DMSP) by other microbes for their survival [17, 71]. To determine if *Prochlorococcus* could fulfill this requirement, we eliminated methionine – the only source of reduced sulfur – from the medium (ProMS -met). Neither strain of *Prochlorococcus* tested (MED4 and MIT9313) could meet SAR11’s need for reduced sulfur (data not shown).

During the initial transfers of this experiment, when the cells were growing on residual methionine, we noticed that the maximum abundance of SAR11 was 1.5-fold lower when co-cultured with *Prochlorococcus* MIT9313 (LLIV clade) vs. growth alone or with *Prochlorococcus* MED4 (HLI clade). Similar results were obtained with another *Prochlorococcus* strain from the LLIV clade (MIT1320, data not shown), suggesting that the LLIV strains may have been competing with SAR11 for a source of reduced sulfur in the Sargasso seawater medium base. Indeed, *Prochlorococcus* populations in the wild have been shown to take up DMSP [72] and, consistent with our observations, the genes required for the transport of DMSP (*proVWX*; the same transporters used for glycine betaine) [69, 73, 74] are found only in members of the LLIV clade of *Prochlorococcus*. To directly address the possibility of competition, we grew SAR11 alone and in co-culture with MIT9313 in a version of ProMS in which the methionine was replaced with an equimolar concentration of DMSP. SAR11 maximal abundances in co-culture with *Prochlorococcus* MIT9313 were 2-fold lower than those in monoculture, providing indirect evidence that MIT9313 may be consuming DMSP in the seawater background, and therefore compete with SAR11 for this reduced sulfur source (Fig. 6c). Thus, not only can the *Prochlorococcus* strains tested not satisfy the reduced sulfur requirement of SAR11, but it is possible that LLIV *Prochlorococcus* may be competing with SAR11 for some forms, such as DMSP, in the wild.

## Summary and concluding remarks

SAR11 grew faster in co-culture with *Prochlorococcus* than in monoculture, while the growth of *Prochlorococcus* was largely unaffected, indicating the production and release of growth factors by *Prochlorococcus* and a commensal relationship (one organism benefits, while the other is not affected) between these organisms under the conditions tested. This relationship became abruptly amensal (one organism is harmed, while the other is not affected) when *Prochlorococcus* cells entered stationary phase, at which point we observed increased DNA fluorescence of SAR11 populations followed by a rapid decline in SAR11 abundance. That stationary phase *Prochlorococcus* cells instead triggered a secondary logarithmic growth phase in diverse copiotrophic bacteria highlights the disparate metabolic capabilities of oligotrophic and copiotrophic marine bacteria and calls for further mechanistic studies. Similarly, the variable growth response of SAR11 in co-culture with *Prochlorococcus* strains belonging to different clades demonstrates the complexity and taxonomic specificity of potential interactions in these autotroph/heterotroph pairings. Furthermore, *Prochlorococcus* MIT9313 enhanced SAR11 growth in co-culture under pyruvate and glycine limited conditions, but had the opposite effect when methionine was limiting, highlighting yet another layer in the complexity of this simple co-culture system. Increased DNA fluorescence per cell was observed for SAR11 populations experiencing pyruvate, glycine, and methionine limiting conditions, consistent with previous observations of cell doublet formation in response to imbalanced nutrient ratios [28]. We also observed increased DNA fluorescence for replete batch cultures in late stationary phase, suggesting this may be a common phenotype for SAR11 HTCC7211 populations prior to cell death.

Collectively, our experimental findings reveal the clade-specific ability of *Prochlorococcus* to increase the growth rate and fulfill some of the unique metabolic requirements of SAR11, while our biogeographic analyses reinforce the notion that these are two of the most ubiquitous and numerically abundant marine bacteria on Earth. Recognizing that laboratory batch cultures cannot mimic environmental conditions, studies of simplified model systems, such as the one described here, lay the foundation for future testing of hypotheses under more realistic conditions in the wild. Now that the steady-state co-culturing of these two iconic marine microbes is possible, follow up studies tracking their transcriptomes, proteomes and metabolomes will help identify the chemical exchanges and physiological dependencies that define their interactions.

## Supplementary information

Supplementary Figure Legends

Supplementary Methods Revised

Table S1 Revised

Figure S1

Figure S2

Figure S3

Figure S4

Figure S5

Figure S6

Figure S7

Figure S8

Table S2 Revised
