# Supplementary material for: Co-culture and biogeography of Prochlorococcus and SAR11
Source: ISME J. 2019 Feb 11;13(6):1506–19. doi: 10.1038/s41396-019-0365-4 (PMC6775983; doi:10.1038/s41396-019-0365-4)
Supplement: Supplementary file 1 — Supplementary Figure Legends [file 41396_2019_365_MOESM1_ESM.docx]

**Supplementary figure legends**

**Figure S1**  Genome phylogenies of *Prochlorococcus* and SAR11. The phylogenies include 135 *Prochlorococcus* and 92 SAR11 isolate, single-cell, and metagenome-assembled genomes. Bootstrap values are represented by filled circles and are sized and colored according to support magnitude. The scale bar is shared for both trees and represents 0.1 amino acid substitutions. The *Prochlorococcus* tree is rooted on *Synechococcus* sp. WH5701 and the SAR11 tree is rooted on TMED13, a metagenome-assembled genome from Tara Oceans distantly related to ‘*Candidatus* Pelagibacter ubique’. Monophyletic clades for both lineages are represented by colored cartoon triangles. Bootstrap support for all clades is greater than 90%, unless specifically denoted with text. Clades utilized in co-culture experiments are marked with a star. Note that the inclusion of new genomes in this study resulted in a polyphyletic origin for the canonical SAR11 clade Ib, therefore we have partitioned clade Ib into 3 monophyletic subclades (Ib.1, Ib.2 and Ib.3).

**Figure S2** Relative abundances of *Prochlorococcus* and SAR11 estimated genome equivalents at different depths and within different ocean basins. Vertical axes represent relative abundance of genome equivalents and horizontal axes represents *Prochlorococcus* (PRO) or SAR11. Horizontal facets bins samples based on ocean region (NA=North Atlantic, SA=South Atlantic, SP=South Pacific, MED=Mediterranean, NP=North Pacific, RS=Red Sea, IND=Indian, HOT=Hawaii Ocean Time-series, BATS=Bermuda Atlantic Time-series) and vertical facets bin by depth ranges. Black horizontal bars represent the median abundance of PRO or SAR11 genome equivalents and points represent individual samples within each region/depth bin. Sample origin (GEOTRACES, Tara Oceans, or HOT and BATS Time-series) are denoted above the horizontal facets.

**Figure S3** Growth rate (a) and maximum cell abundance (b) of SAR11 (*Pelagibacterales sp.* HTCC7211) monocultures grown in various artificial and natural seawater-based media types. AMS1^*^ is the artificial medium designed by Carini et al. for SAR11 [1] supplemented with pyruvate (50 µM), glycine (50 µM) and methionine (10 µM). ProMS^*^ is natural seawater based Pro99 medium [2] supplemented with pyruvate (50 µM), glycine (50 µM), methionine (10 µM) and the vitamin mix used for AMS1. ProMS^*^ reduced is Pro99 medium supplemented with pyruvate (2.5 µM), glycine (2.5 µM), methionine (0.5 µM) and the vitamin mix used for AMS1. ProMS is the medium designed for the co-culture of *Prochlorococcus* and SAR11 (see Table S1).

**Figure S4** Cell abundance as a function of time for SAR11 (*Pelagibacterales sp.* HTCC7211) (black line) and *Prochlorococcus* strain MED4 (gray line) in a semi-continuous batch co-culture maintained in ProMS medium. Dilution frequency and volume were dictated by *Prochlorococcus* cell density (see main text). A subset of this data set is plotted in Fig. 3a to more clearly depict the phase relationship between the strains.

**Figure S5** Growth of *Prochlorococcus* strains MED4 (a), MIT9312 (b), MIT0801 (c) and MIT9313 (d) in co-culture with SAR11 (dashed gray lines) compared to the growth of the *Prochlorococcus* strains in monoculture (black lines). Open circles represent the mean (± s.d.) of biological triplicates. Error bars are smaller than the size of the symbols where not visible.

**Figure S6** Flow cytometry signatures of SAR11 (red) and either *Prochlorococcus* strain MED4 or MIT9313 (green) in co-culture. MED4 and MIT9313 began to slow their growth rates and transition into stationary phase on day 12 and day 10, respectively. There were no detectable SAR11 populations remaining on day 15. Histograms display an increase in the DNA fluorescence of SAR11 populations in co-culture (pink) compared to monoculture (gray) beginning on either day 12 or day 10.

**Figure S7** Growth of *Prochlorococcus* strains MIT1314 (HLII clade; a) and MIT1327 (LLIV clade; b) in co-culture with sympatric copiotrophic bacteria: *Thalassospira sp.* MIT1351 (dashed gray stars), *Roseobacter sp.* MIT1352 (dashed gray circles) and *Marinobacter sp.* MIT1353 (dashed gray triangles) compared to the growth of *Prochlorococcus* strains in monoculture (black lines). Open symbols represent the mean (± s.d.) of biological triplicates. Error bars are smaller than the size of the symbols where not visible.

**Figure S8** Relative abundances of *Prochlorococcus* (green) and SAR11 (red) based on estimated genome equivalents (GE) or based on the total fraction of mapped reads. Each point represents a metagenome sample. The thin solid black line represents the 1:1 ratio of equal proportion of mapped reads to GE. In most cases, both *Prochlorococcus* and SAR11 estimates fall below this line, indicating that relative abundances determined by total mapped reads underestimate the relative abundances of discrete genomes in each sample. Thick black lines are linear models fit to the relative abundance of *Prochlorococcus* (dashed) and SAR11 (solid).

**References**

1. Carini P, Steindler L, Beszteri S, Giovannoni SJ. Nutrient requirements for growth of the extreme oligotroph ‘*Candidatus* Pelagibacter ubique’ HTCC1062 on a defined medium. ISME J 2013;7:592–602.

2. Moore LR, Coe A, Zinser ER, Saito MA, Sullivan MB, Lindell D, et al. Culturing the marine cyanobacterium *Prochlorococcus*. Limnol. Oceanogr. Methods 2007;5:353–62.
