# Supplementary material for: Co-culture and biogeography of Prochlorococcus and SAR11
Source: ISME J. 2019 Feb 11;13(6):1506–19. doi: 10.1038/s41396-019-0365-4 (PMC6775983; doi:10.1038/s41396-019-0365-4)
Supplement: Supplementary file 2 — Supplementary Methods Revised [file 41396_2019_365_MOESM2_ESM.docx]

**Supplementary Methods**

This file contains expanded materials and methods

**Isolation of sympatric *Prochlorococcus* and heterotrophic bacteria**

We had hoped to isolate sympatric *Prochlorococcus* and oligotrophic (k-selected) heterotrophic pairs (e.g. SAR11), but we were unsuccessful. We succeeded, however, in isolating new *Prochlorococcus* strains, along with sympatric copiotrophic bacteria, from the N. Pacific (Table 1). The latter were chosen to represent a broad phylogenetic range of heterotrophic bacteria, including both alpha- and gammaproteobacteria. *Prochlorococcus* MIT1314 (HLII clade) and MIT1327 (LLIV clade) were isolated using established enrichment protocols [1] with some modifications [2], and rendered axenic via high-throughput dilution-to-extinction culturing as previously described [3]. The purity of these isolates was confirmed by flow cytometry and a suite of purity test broths: ProAC, ProMM and MPTB [3]. Sympatric heterotrophic bacteria strains MIT1351 (*Thalassospira*), MIT1352 (*Roseobacter*) and MIT1353 (*Marinobacter*) were isolated from the *Prochlorococcus* enrichment that generated the two new *Prochlorococcus* strains. The *Thalassospira* strain was isolated via high-throughput dilution-to-extinction culturing as previously described [4] using a sterile Sargasso seawater-based medium amended with ammonium (1 µM) and phosphate (0.1 µM). Dilution plates were maintained at 23 ^o^C under constant illumination (3 µmol photons m^-2^ s^-1^). The *Roseobacter* and *Marinobacter* strains were isolated as colonies on soft agar (0.3%) pour plates consisting of Pro99 medium amended with pyruvate (0.05%) and TAPS buffer (3.75 mM). Agar plates were maintained at 23 ^o^C under constant illumination (1 to 6 µmol photons m^-2^ s^-1^). Colonies with distinct morphologies were picked and passed at least 3 times before transfer into liquid Pro99 medium. Purity of the heterotrophic bacteria strains was assessed by flow cytometry and genomic sequencing. The strains were maintained on Sargasso seawater-based Pro99 medium in acid-washed autoclaved borosilicate glass tubes at 23 ^o^C under constant illumination (11 µmol photons m^-2^ s^-1^) prior to co-culturing as, unlike SAR11, they could grow in unamended Pro99.

**Genome sequencing**

Genomes of *Prochlorococcus* MIT1314 and heterotrophic bacteria strains MIT1351, MIT1352 and MIT1353 were sequenced from genomic DNA obtained from 35 to 50 ml laboratory cultures. Cells were concentrated by centrifugation (10 000 *g* for 10 min) and genomic DNA was isolated using the MasterPure complete DNA and RNA purification kit (Epicentre). 2 µg of high-molecular weight genomic DNA was sheared, and 625 – 650 ng of sheared DNA was used to construct libraries using a standard SMRTbell Template Prep Kit 1.0 and Sequencing Primer v3 (Pacific Biosciences) with the addition of a gap repair and end polishing step to ensure closed single-stranded template. Quality control analysis was performed before and after library construction using a Q-bit, NanoVue, and an Advanced Analytics DNA Fragment Analyzer, and DNA was quantified using PicoGreen assays. The average insert range for completed libraries was 21 – 28 kb. DNA libraries were sequenced using P6 chemistry on a Pacific Biosystems RS II instrument with a 240 min data collection time per SMRTcell. Data processing using SMRT Analysis 2.0 was performed to remove linkers and trim rear ends. *De novo* assembly into a single contig was performed using the SMRT Portal software and the RS_HGAP_Assembly.2 protocol. The mean read quality score for each assembly was between 0.85 and 0.86 and coverage ranged from 73X to 168X. Genomes were circularized using the Geneious sequence analysis package (V7.1, Biomatters) prior to polishing using Quiver and the RS_Resequencing.1 protocol within the SMRT Portal software to obtain more accurate consensus calls. Closed circular genomes were deposited in the Joint Genome Institute’s Integrated Microbial Genomes (IMG) system and annotated using the IMG Annotation Pipeline version 4 [5,6]. Library construction and sequencing was performed at the University of Massachusetts Medical School Deep Sequencing and Molecular Biology Core Laboratories.

**Data sources**

Reference genomes used in this study were downloaded as nucleotide assemblies from NCBI GenBank or Refseq and the Prokka tool [7] was used to call genes and produce annotations for all genomes. We constructed a custom nonredundant 56 million sequence reference database of microbial proteins for use with the protein homology classifier Kaiju [8]. To ensure maximum representation of marine bacteria, archaea, and microbial eukaryotes, we included translated genes/transcripts from 5 397 representative “specI” species clusters from the proGenomes database [9]; 113 transcriptomes from the Marine Microbial Eukaryote Transcriptome Sequencing Project (MMETSP) [10]; 10 509 metagenome assembled genomes from the Tara Oceans expedition [11,12], the Red Sea [13], the Baltic Sea [14], and other aquatic and terrestrial sources [15]; 994 isolate genomes from the Genomic Encyclopedia of Bacteria and Archaea [16]; 7 492 viral genomes from NCBI RefSeq [17]; 786 bacterial and archaeal genomes from MarRef [18]; and 677 marine single cell genomes [19]. Metagenomic read files to be annotated from the Tara Oceans project [20-22] were downloaded from the European Nucleotide Archive. The remaining 606 metagenomes were recently described [23] and are available from NCBI.

**Metagenome quality control**

We preprocessed raw metagenome reads using the bbtools software suite (BBMap V37.90; [24]). Briefly, we trimmed reads of Illumina adapters using kmer matches (k=23) to known adapter sequences from the 5’ end of the read. We required a Maximum Hamming distance for reference kmers of 1, and we discarded reads with 3 or more ‘Ns’ or with an average quality score of less than Q20. We further trimmed overlapping reads based on insert size (tbo=t) if adapter kmers were not identified and both reads were trimmed to the minimum length of the read pair. Finally, we discarded any reads shorter than 60 bp after all adapter trimming steps. We then discarded reads containing common sequencing artifacts using kmer matches (k=31) to known Illumina sequence artifacts provided in the bbtools software suite. Reads were merged using the bbmerge tool with default parameters, a minimum insert size of 35 bp, and a minimum overlapping sequence of 12 bp. We retained all unmerged and orphaned reads that passed quality control for downstream annotation and analysis.

**Genome quality control**

Because our goal was to annotate *Prochlorococcus* and SAR11 reads from shotgun metagenomes, we needed to ensure taxonomic fidelity of all genomes used as annotation references. We determined an initial genome taxonomy for all genome bins used to construct our reference genome database using checkM (V1.0.11) with the default lineage workflow [25] and ensured that all genome bins met the completion/contamination thresholds outlined in prior studies [12,15]. We then selected genomes with a taxonomic assignment of *Prochlorococcus* or SAR11 (*Pelagibacterales* order) for further analysis and confirmed taxonomic assignments using blast matches to known *Prochlorococcus* ITS sequences and by matching 16S sequences of SAR11 to the SILVA database [26]. To refine our estimates of completeness/contamination of *Prochlorococcus* genome bins, we created a custom set of 503 single copy genes from closed, isolate-derived *Prochlorococcus* genomes [27] for quality assessments with checkM. For SAR11, we used the checkM taxonomic-specific workflow with the order *Pelagibacterales*. After the custom checkM quality control, we excluded genomes from downstream phylogenetic analysis that had an estimated quality less than 30, defined as %completeness – 5 X %contamination.

**Metagenome read classification**

We taxonomically classified metagenome reads using the short read classifier Kaiju v1.6.0 [8] with a custom, curated reference database comprised of approximately 26 000 bacterial, archaeal, viral, and eukaryotic proteomes (methods section “Data Sources”). The taxonomic composition of the database was intended to predominantly reflect that of the marine environment, while minimizing (but not excluding) the representation of clinical, industrial, and terrestrial host-associated samples, which greatly increased the classification speed of the more than 800 metagenomes analyzed in this study. We excluded sequences from our custom database with lengths less than 20 and greater than 20 000 amino acids, removed non-standard amino acid residues, and condensed redundant protein sequences to a single representative sequence to which we assigned a lowest common ancestor taxonomy identifier from the NCBI taxonomy database [28]. We used Kaiju in ‘mem’ mode with a minimum BLOSUM62 score of 65 for fragment matching and a SEG filter to mask low complexity sequences. We allowed for up to three amino acid substitutions in each match alignment and excluded matches with a Kaiju “expect value” less than 0.05. Using this curated database, the majority of reads in our study (54% ± 10%) could be classified across all metagenomes.

**Relative taxonomic abundance from total reads and marker genes**

We used the abundance of single-copy, universal microbial marker genes [29-31] to estimate taxonomic abundances based on total counts of taxonomically recruited reads. In this approach, universal genes present in equal copy number in all bacteria and archaea are quantified and taxonomically classified, which addresses potential systematic biases introduced by changes in average genome size (AGS) and/or taxonomic composition. Thus, the abundance of different taxonomic groups can be expressed in terms of discrete ‘genomic equivalents’ rather than individual reads, which is independent of AGS and more intuitively related to the abundance of individual taxa. *Prochlorococcus* (1.6-2.5 Mb) and SAR11 (1.2-1.4 Mb) both have small genomes, thus their relative abundance estimated from total recruited reads could underestimate their true abundance, while overestimating the abundance of microbes with larger genomes. Indeed, we found that relative read abundance and estimated relative genome equivalents (using MicrobeCensus V1.1.1; [32], see below) of *Prochlorococcus* and SAR11 had a strong linear relationship, while read counts alone systematically underestimated the relative abundance of either organism, especially when they constituted a quarter or more of the identifiable bacteria and archaea in a given sample (Fig. S8). This finding supports previous work demonstrating that AGS differences between microbial groups can distort relative abundance estimates based on total read classification depending on the degree of variability and the relative contribution of differently sized genomes to the total genome pool in a microbial community [32,33].

**Relative abundance estimates of *Prochlorococcus* and SAR11 using genome equivalents**

To address potential artifacts related to genome size, we used a hybrid marker gene/total read classification approach to estimate genome equivalents for taxonomically resolved read groups in each metagenome. We first classified all reads in each metagenome using Kaiju and the custom database described above, and then pulled reads mapping to *Prochlorococcus* or SAR11, and all reads annotated as bacteria and archaea, while excluding reads annotated as microbial eukaryotes, viruses, or those with no classification. We then quantified marker genes within each taxonomically partitioned read pool using the MicrobeCensus (V1.1.1) algorithm [32]. MicrobeCensus quantifies 30 well-characterized single-copy microbial marker genes using sensitive gene family-specific sequence similarity thresholds, but without any taxonomic distinction. Then the algorithm uses proportionality constants and weights for each gene family to relate counts to the relative abundance for each marker gene. This relative abundance is multiplied by the inverse of linear model coefficients for each gene family to obtain an estimate of AGS for each marker family, the weighted average of which becomes the total AGS. In each step, all model coefficients, thresholds, weights and proportionality constants have been estimated from simulated metagenomes of defined taxonomic abundance and composition. The number of genome equivalents within a taxonomically resolved read pool is calculated by the total number of bases divided by the estimated AGS. In this way, we obtained information about AGS and the number of genome equivalents for the genus-integrated *Prochlorococcus* and order-integrated SAR11 (*Pelagibacterales*) populations in each metagenome. The mean AGS was 1.46 ± 0.24 Mb for *Prochlorococcus* and 1.2 ± 0.10 Mb for SAR11 across all samples, which agrees well with the genome sizes of sequenced isolates from these groups [27,34]. We then compared the proportion of *Prochlorococcus* or SAR11 genome equivalents to the total number of bacterial and archaeal genome equivalents detected in each sample to determine relative abundances.

The relative abundance of any taxonomic group in a metagenome can be distorted by the proportion of unclassified or unknown reads, particularly if this unclassified proportion varies randomly across samples/environments/conditions being compared. Although we constructed a taxonomically diverse and comprehensive reference database specifically tailored to the marine environment, we undoubtedly missed some of the microbial diversity present in each sample. This is a source of error endemic to any microbiome study, and metagenomic studies in under-sampled environments (e.g. marine, soil) typically have significantly lower read recruitment rates than studies in well-characterized systems (e.g. human or other host-associated). The majority of reads in our study (54% ± 10%) could be classified across all metagenomes, with the largest numbers of unclassified reads found in GEOTRACES samples, particularly in neritic zones and from latitudes greater than 30 degrees N/S. The GA10 transect in particular displayed the lowest total recruitment, as well as the lowest specific recruitment to *Prochlorococcus* and SAR11. In contrast, samples from the Tara oceans project, particularly those from equatorial waters, had the highest classification rates - often approaching 80%. This classification discrepancy between the two sequencing projects is likely because the Tara samples used here were prefiltered to exclude larger eukaryotes, which are often poorly represented in reference database [22], whereas the GEOTRACEs samples were not prefiltered [23]. Additionally, our reference database included over 2 500 metagenome assembled genomes derived from the Tara oceans prokaryotic size fractions [11,12], which we would expect to strongly recruit metagenomic reads from Tara samples.

We estimated the abundance of marker genes (using MicrobeCensus) in the unclassified read fraction (from Kaiju) for 100 randomly selected metagenomes, and found that the number of genome equivalents ‘missed’ in this unclassified fraction averaged < 5% of the total number of genome equivalents estimated for all bacteria and archaea in each metagenome, suggesting that our unclassified read pools are predominantly comprised of novel, unknown gene families rather than universal single-copy genes. Therefore, we expect our genome equivalent estimates to be robust to variability in read recruitment rates, however it is possible that our methodological approach may have missed some small fraction of highly novel bacterial/archaeal genome equivalents, resulting in a slight overestimation of the relative contribution of SAR11 and *Prochlorococcus* to the total prokaryotic community.

**Phylogenetic inference**

The *Prochlorococcus* phylogeny (Fig. S1a) includes 135 isolate, single-cell, and metagenome-assembled genomes (estimated > 90% complete), and the SAR11 phylogeny (Fig. S1b) includes 92 isolate, single-cell, and metagenome-assembled genomes (estimated > 70% complete). These completeness thresholds were selected as a compromise between maximum completeness and phylogenetic diversity of the available genomes. Each phylogeny is based on a concatenated protein multiple sequence alignment of 120 taxonomically conserved single copy marker genes [15,35]. We used GTDB-Tk (V0.0.7; https://github.com/Ecogenomics/GTDBTk) with default settings to generate the alignments using HMMER V3.1b2; http://hmmer.org/). We pruned/trimmed both alignments to remove genomes that filled fewer than 50% of alignment positions in order to remove columns represented by fewer than 50% of all taxa and to remove columns with no single amino acid residue occurring at a frequency greater than 25%. We further trimmed the alignments using trimAl with the automated *-gappyout* option to trim columns based on their gap distribution. Genomes were pruned from a starting tree to allow a maximum average distance to closest leaf (ADCL) of 0.003 to reduce the phylogenetic redundancy within the overall phylogeny [36]. We constructed phylogenies using Maximum Likelihood inference with multithreaded RAxML (V8.2.9; [37]) using the GAMMA model of rate heterogeneity, empirically determined base frequencies, and the LG substitution model [38] (PROTGAMMALGF). Branch support is based on 250 resampled bootstrap trees.

**References**

1. Moore LR, Coe A, Zinser ER, Saito MA, Sullivan MB, Lindell D, et al. Culturing the marine cyanobacterium *Prochlorococcus*. Limnol. Oceanogr. Methods 2007;5:353–62.

2. Cubillos-Ruiz A, Berta-Thompson JW, Becker JW, van der Donk WA, Chisholm SW. Evolutionary radiation of lanthipeptides in marine cyanobacteria. Proc. Natl. Acad. Sci. U.S.A. 2017;114:E5424–33.

3. Berube PM, Biller SJ, Kent AG, Berta-Thompson JW, Roggensack SE, Roache-Johnson KH, et al. Physiology and evolution of nitrate acquisition in *Prochlorococcus*. ISME J 2014;9:1195–207.

4. Connon SA, Giovannoni SJ. High-throughput methods for culturing microorganisms in very-low-nutrient media yield diverse new marine isolates. Applied and Environmental Microbiology 2002;68:3878–85.

5. Huntemann M, Ivanova NN, Mavromatis K, Tripp HJ, Paez-Espino D, Palaniappan K, et al. The standard operating procedure of the DOE-JGI Microbial Genome Annotation Pipeline (MGAP v.4). Standards in Genomic Sciences 2016;10:86.

6. Markowitz VM, Chen I-MA, Palaniappan K, Chu K, Szeto E, Pillay M, et al. IMG 4 version of the integrated microbial genomes comparative analysis system. Nucleic Acids Res. 2013;42:D560–7.

7. Seemann T. Prokka: rapid prokaryotic genome annotation. Bioinformatics 2014;30:2068–9.

8. Menzel P, Ng KL, Krogh A. Fast and sensitive taxonomic classification for metagenomics with Kaiju. Nat Comms 2016;7:11257.

9. Mende DR, Letunic I, Huerta-Cepas J, Li SS, Forslund K, Sunagawa S, et al. proGenomes: a resource for consistent functional and taxonomic annotations of prokaryotic genomes. Nucleic Acids Res. 2017;45:D529–34.

10. Keeling PJ, Burki F, Wilcox HM, Allam B, Allen EE, Amaral-Zettler LA, et al. The Marine Microbial Eukaryote Transcriptome Sequencing Project (MMETSP): Illuminating the functional diversity of eukaryotic life in the oceans through transcriptome sequencing. PLoS Biol 2014;12:e1001889.

11. Tully BJ, Sachdeva R, Graham ED, Heidelberg JF. 290 metagenome-assembled genomes from the Mediterranean Sea: a resource for marine microbiology. PeerJ 2017;5:e3558.

12. Tully BJ, Graham ED, Heidelberg JF. The reconstruction of 2,631 draft metagenome-assembled genomes from the global oceans. Sci. Data 2018;5:170203–8.

13. Haroon MF, Thompson LR, Parks DH, Hugenholtz P, Stingl U. A catalogue of 136 microbial draft genomes from Red Sea metagenomes. Sci. Data 2016;3:160050.

14. Hugerth LW, Larsson J, Alneberg J, Lindh MV, Legrand C, Pinhassi J, et al. Metagenome-assembled genomes uncover a global brackish microbiome. Genome Biology 2015;16:279.

15. Parks DH, Rinke C, Chuvochina M, Chaumeil P-A, Ben J Woodcroft, Evans PN, et al. Recovery of nearly 8,000 metagenome-assembled genomes substantially expands the tree of life. Nature Microbiology 2017;2:1533–42.

16. Mukherjee S, Seshadri R, Varghese NJ, Eloe-Fadrosh EA, Meier-Kolthoff JP, Göker M, et al. 1,003 reference genomes of bacterial and archaeal isolates expand coverage of the tree of life. Nature Biotechnology 35:676–83.

17. Haft DH, DiCuccio M, Badretdin A, Brover V, Chetvernin V, O’Neill K, et al. RefSeq: an update on prokaryotic genome annotation and curation. Nucleic Acids Res. 2017;46:D851–60.

18. Klemetsen T, Raknes IA, Fu J, Agafonov A, Balasundaram SV, Tartari G, et al. The MAR databases: development and implementation of databases specific for marine metagenomics. Nucleic Acids Res. 2017;46:D692–9.

19. Berube PM, Biller SJ, Hackl T, Hogle SL, Satinsky BM, Becker JW, et al. Single cell genomes of *Prochlorococcus*, *Synechococcus*, and sympatric microbes from diverse marine environments. Sci. Data 2018;5:180154.

20. Karsenti E, Acinas SG, Bork P, Bowler C, de Vargas C, Raes J, et al. A holistic approach to marine eco-systems biology. PLoS Biol 2011;9:e1001177.

21. Sunagawa S, Coelho LP, Chaffron S, Kultima JR, Labadie K, Salazar G, et al. Structure and function of the global ocean microbiome. Science 2015;348:6237.

22. Pesant S, Not F, Picheral M, Kandels-Lewis S, Le Bescot N, Gorsky G, et al. Open science resources for the discovery and analysis of Tara Oceans data. Sci. Data 2015;2:150023.

23. Biller SJ, Berube PM, Dooley K, Williams M, Satinsky B, Hackl T, et al. Marine microbial metagenomes sampled across space and time. Sci. Data 2018;5:180176.

24. Bushnell B, Rood J, Singer E. BBMerge – Accurate paired shotgun read merging via overlap. PLoS ONE 2017;12:e0185056–15.

25. Parks DH, Imelfort M, Skennerton CT, Hugenholtz P, Tyson GW. CheckM: assessing the quality of microbial genomes recovered from isolates, single cells, and metagenomes. Genome Research 2015;25:1043–55.

26. Quast C, Pruesse E, Yilmaz P, Gerken J, Schweer T, Yarza P, et al. The SILVA ribosomal RNA gene database project: improved data processing and web-based tools. Nucleic Acids Res. 2013;41:D590–6.

27. Biller SJ, Berube PM, Berta-Thompson JW, Kelly L, Roggensack SE, Awad L, et al. Genomes of diverse isolates of the marine cyanobacterium *Prochlorococcus*. Sci. Data 2014;1:140034–11.

28. Federhen S. The NCBI Taxonomy database. Nucleic Acids Res. 2011;40:D136–43.

29. Segata N, Waldron L, Ballarini A, Narasimhan V, Jousson O, Huttenhower C. Metagenomic microbial community profiling using unique clade-specific marker genes. Nat Meth 2012;9:811–4.

30. Nayfach S, Rodriguez-Mueller B, Garud N, Pollard KS. An integrated metagenomics pipeline for strain profiling reveals novel patterns of bacterial transmission and biogeography. Genome Research 2016;26:1612–25.

31. Boyd JA, Woodcroft BJ, Tyson GW. GraftM: a tool for scalable, phylogenetically informed classification of genes within metagenomes. Nucleic Acids Res. 2018;46:e59–9.

32. Nayfach S, Pollard KS. Average genome size estimation improves comparative metagenomics and sheds light on the functional ecology of the human microbiome. Genome Biol. 2015;16:59–18.

33. Beszteri B, Ben Temperton, Frickenhaus S, Giovannoni SJ. Average genome size: a potential source of bias in comparative metagenomics. ISME J 2010;4:1075–7.

34. Grote J, Thrash JC, Huggett MJ, Landry ZC, Carini P, Giovannoni SJ, et al. Streamlining and core genome conservation among highly divergent members of the SAR11 clade. mBio 2012;3:e00252–12.

35. Parks DH, Chuvochina M, Waite DW, Rinke C, Skarshewski A, Chaumeil P-A, et al. A standardized bacterial taxonomy based on genome phylogeny substantially revises the tree of life. Nature Publishing Group 2018;:1–14.

36. Matsen FA IV, Gallagher A, McCoy CO. Minimizing the average distance to a closest leaf in a phylogenetic tree. Systematic Biology 2013;62:824–36.

37. Stamatakis A. RAxML-VI-HPC: maximum likelihood-based phylogenetic analyses with thousands of taxa and mixed models. Bioinformatics 2006;22:2688–90.

38. Le SQ, Gascuel O. An improved general amino acid replacement matrix. Molecular Biology and Evolution 2008;25:1307–20.
