# Supplementary material for: Co-culture and biogeography of Prochlorococcus and SAR11
Source: ISME J. 2019 Feb 11;13(6):1506–19. doi: 10.1038/s41396-019-0365-4 (PMC6775983; doi:10.1038/s41396-019-0365-4)
Supplement: Supplementary file 3 — Table S1 Revised [file 41396_2019_365_MOESM3_ESM.docx]

**Table S1** Composition of media used for co-culture of *Prochlorococcus* and SAR11. Filter-sterilized surface seawater from the Sargasso Sea served as the base for all media types. ProMS and ProMC were created, through trial and error, based on Pro99 [1] and AMS1 [2].

| Component | ProMS | ProMC | ProMS  -gly | ProMS  -met |
| --- | --- | --- | --- | --- |
|  |  |  |  |  |
| *Macronutrients* |  |  |  |  |
| NH_4_Cl | 800 µM | 800 µM | 800 µM | 800 µM |
| NaH_2_PO_4_·H_2_O | 50 µM | 50 µM | 50 µM | 50 µM |
|  |  |  |  |  |
| *Trace Metals* |  |  |  |  |
| Na_2_EDTA·2H_2_O | 1.17 µM | 1.17 µM | 1.17 µM | 1.17 µM |
| FeCl_3_·6H_2_O | 1.17 µM | 1.17 µM | 1.17 µM | 1.17 µM |
| MnCl_2_·4H_2_O | 90 nM | 90 nM | 90 nM | 90 nM |
| ZnSO_4_·7H_2_O | 8 nM | 8 nM | 8 nM | 8 nM |
| CoCl_2_·6H_2_O | 5 nM | 5 nM | 5 nM | 5 nM |
| Na_2_MoO_4_·2H_2_O | 3 nM | 3 nM | 3 nM | 3 nM |
| Na_2_SeO_3_ | 10 nM | 10 nM | 10 nM | 10 nM |
| NiCl_2_·6H_2_O | 10 nM | 10 nM | 10 nM | 10 nM |
|  |  |  |  |  |
| *Vitamins* |  |  |  |  |
| B_1_ | 120 nM | 6 µM | 120 nM | 120 nM |
| B_3_ | 16 nM | 800 nM | 16 nM | 16 nM |
| B_5_ | 8.5 nM | 425 nM | 8.5 nM | 8.5 nM |
| B_6_ | 10 nM | 500 nM | 10 nM | 10 nM |
| B_7_ | 80 pM | 4 nM | 80 pM | 80 pM |
| B_9_ | 80 pM | 4 nM | 80 pM | 80 pM |
| B_12_ | 14 pM | 700 pM | 14 pM | 14 pM |
| Myo-inositol | 120 nM | 6 µM | 120 nM | 120 nM |
| 4-Aminobenzoic acid | 1.2 nM | 60 nM | 1.2 nM | 1.2 nM |
|  |  |  |  |  |
| Sodium pyruvate | 1 µM |  | 1 µM | 1 µM |
| Glycine | 1 µM | 50 µM |  | 1 µM |
| L-Methionine | 200 nM | 10 µM | 200 nM |  |
|  |  |  |  |  |

**References**

1. Moore LR, Coe A, Zinser ER, Saito MA, Sullivan MB, Lindell D, et al. Culturing the marine cyanobacterium *Prochlorococcus*. Limnol. Oceanogr. Methods 2007;5:353–62.

2. Carini P, Steindler L, Beszteri S, Giovannoni SJ. Nutrient requirements for growth of the extreme oligotroph “*Candidatus* Pelagibacter ubique” HTCC1062 on a defined medium. ISME J 2013;7:592–602.
